# “Then I went to a hospital abroad”: acknowledging implications of stakeholders’ differing risk understandings related to use of complementary and alternative medicine in European health care contexts

**DOI:** 10.1186/s12906-019-2499-3

**Published:** 2019-04-30

**Authors:** Anita Salamonsen, Solveig Wiesener

**Affiliations:** 1 Regional Centre for Child and Youth Mental Health and Child Welfare (RKBU North), Faculty of Health Sciences, UiT The Arctic University of Norway, 9037 Tromsø, Norway; 2 The National Research Center in Complementary and Alternative Medicine (NAFKAM), Faculty of Health Sciences, Department of Community Medicine, UiT The Arctic University of Norway, 9037 Tromsø, Norway

**Keywords:** Complementary and alternative medicine, Traditional and complementary medicine, Refusal of conventional treatment, Risk definitions, Risk perceptions, Risk understandings: risk behaviors, Patient safety, Quality in health care, Health policy-making, Decision making, Person-centeredness, Doctor-patient communication, Risk communication, Risk ontology, Regulation, risk governance, Norway, Europe

## Abstract

**Background:**

Complementary and alternative medicine (CAM) is a rather novel issue within public healthcare and health policy-making. CAM use in Europe is widespread, patient-initiated, and patient-evaluated, and the regulation across countries has been evaluated as disharmonized. CAM users are left in an uncertain position, and patient safety may be threatened. How “risk” is understood by individuals in health policy-making and clinical encounters involving the use of CAM has not yet been much debated. The aim of this article is to explore and discuss the existence and possible consequences of differing risk understandings among stakeholders maneuvering in the complex landscape of CAM practice and CAM regulation contextualized by European public healthcare systems.

**Methods:**

Qualitative data were derived from two studies on CAM in European healthcare contexts. Findings from the EU project CAMbrella on legislation and regulation of CAM were mixed with data from an interview study exploring risk understandings, communication, and decision-making among Scandinavian CAM users and their doctors. In a secondary content analysis, we constructed the case *Sara* as a typology to demonstrate important findings with regard to risk understandings and patient safety involving European citizens’ use of CAM in differing contexts.

**Results:**

By combining and comparing individual and structural perspectives on risk and CAM use, we revealed underexplored gaps in risk understandings among individuals involved in European CAM regulation and legislation, and between CAM users and their medical doctors. This may cause health risks and uncertainties associated with CAM use and regulation. It may also negatively influence doctor-CAM user communication and CAM users’ trust in and use of public healthcare.

**Conclusion:**

Acknowledging implications of stakeholders’ differing risk understandings related to CAM use and regulation may positively influence patient safety in European healthcare. Definitions of the concept of risk should include the factors uncertainty and subjectivity to grasp the full picture of possible risks associated with the use of CAM. To transform the findings of this study into practical settings, we introduce sets of questions relevant to operationalize the important question “What is risk?” in health policy-making, clinical encounters and risk research involving European patients’ use of CAM.

## Background

There is no agreed definition of the concept of risk in contemporary risk research, and various understandings may have different strengths and weaknesses [1, 2]. Complementary and alternative medicine (CAM) is a rather novel field within risk research and health policy-making. What constitutes CAM varies with a number of factors and contexts, and definitions of CAM change over time as the borders between public healthcare and CAM are constantly changing. The Cochrane Collaboration has defined CAM as “… a broad domain of healing resources that encompasses all health systems, modalities and practices, and their accompanying theories and beliefs, other than those intrinsic to the politically dominant health system” [3], p. 693. Norway is one of very few countries where CAM is legally defined. The Norwegian Act No. 64 of 27 June 2003 relating to the alternative treatment of disease, illness, and so forth [4] defines CAM as:

*… health related treatment which is practiced outside the established health services and which is not practiced by authorized health personnel. However, treatment practiced within the scope of the established health services or by authorized health personnel is also covered by the term alternative treatment when the methods used are essentially methods that are used outside the established health services.*

Recent studies show a widespread and continuing use of CAM among European citizens, although prevalence of use varies greatly by country [5–11]. Reasons for use are complex, but is often linked to health-related and sociodemographic determinants such as gender, income and self-defined health status [11]. In the Scandinavian countries, for instance, the prevalence of CAM use is higher among middle-aged women with higher education, and people with poor self-reported health [12]. CAM users may be considered deviant and noncompliant because they challenge the rationality of medical advice [13–15], and they report a significantly “lower level of confidence in the efficacy of conventional medicine”than non-users [16]. However, most CAM users do not leave the public healthcare system despite negative experiences that sometimes function as push-factors for their decision to use CAM [11–14, 17, 18]. They uphold a relationship to public healthcare, and studies show that most CAM users want to discuss possible benefits and risks associated with their CAM use with their conventional healthcare providers [14, 19]. Despite this, an indirect risk related to CAM use is that as many as 50% of CAM users actually do not disclose their use of CAM in clinical settings in public healthcare, often because they fear that raising this issue may negatively affect the patient-doctor relation [14, 20, 21].

### Diverging risk understandings

We know from other fields of risk research that diverging understandings of what risk is exist both between policy makers, healthcare professionals and the public, and that this may affect the way risk is handled [1, 2, 22, 23]. Despite the widespread use of CAM, there has so far been little attention towards the importance of possible differing risk understandings among stakeholders. This may influence decision-making and patient safety in this field of European healthcare. A main question in ongoing debates about risk understandings within other fields of risk research is whether the concept of risk can escape an element of subjectivity. In other words, can risk be considered as an ontological, objective fact to be measured and explained, or not [1, 2, 22, 23]? Based on the debate in central scientific risk journals, a hermeneutic understanding of risk as a social construct has been introduced as a supplement to the traditionally dominant understanding of risk as an objective fact that we also recognize in conventional health care settings. CAM users experience benefits from CAM that seem to elude the evidence of efficacy and risk factors generated in RCT research in their decision-making processes [24–26]. The central risk researchers Aven, Renn, and Rosa [27] in 2011 identified two prevailing definitions of risk that we find to be a relevant approach to risk understandings related to CAM regulation, risk communication in clinical settings, and European CAM users’ decision-making:

(i) *Risk is a situation or event where something of human value (including humans themselves) is at stake and where the outcome is uncertain*
(ii) *Risk is an uncertain consequence of an event or an activity with respect to something that humans value* ([27], p., 1074)

In this perspective, risk does include uncertainty and subjective elements, factors that have been identified as important in understanding modern patients’ use of CAM [24–26, 28, 29]. Risk understandings among different individuals in the field of CAM may be interpreted based on a variety of contextual factors involving individual experience, cultural beliefs, social practices, attitudes, and values.

### Research initiatives leading to this paper

The authors of this article have previously conducted two different qualitative studies where information on such various risk understandings associated with the use of CAM in Europe turned out to be important empirical patterns. A study among Scandinavian CAM users and their physicians in public healthcare revealed rather fundamental gaps in risk understandings. These gaps were found to influence decision-making and communication in ways that may negatively influence patient safety for CAM users who are also patients in Scandinavian public healthcare systems [14, 15, 28, 29]. The EU-study CAMbrella, dealing with legislation and regulation of CAM in 39 European countries, revealed no harmony in legislation, regulation, CAM definitions, terminology, treatments, providers’ skills or regulatory systems across the 39 countries. On a structural level, comparable CAM professionals and treatments are defined as conventional care within the public healthcare system in one country and as CAM in other countries, depending on national regulation [30–34]. The CAMbrella data have so far been analyzed from different angles with respect to risk theories and risk understandings. Wiesener [33] discusses whether CAM regulation in Europe is in accordance with current risk theories related to health governance and patient safety, whilst Wiesener and colleagues [34] discuss possible risk understandings among European health policy-makers on a structural level. In this article, we explore and discuss differing risk understandings on an individual level among persons seeking healthcare, healthcare professionals, CAM providers and representatives for the health authorities. We combine the perspectives and findings from our earlier studies in an analysis of differing risk understandings and their possible implications for patient safety across structural and individual levels of communication and decision-making.

### Aims and research questions

The overall aim of this article is to contribute to an awareness of the possible existence and importance of differing risk understandings with regard to patients’ use of CAM, both in clinical encounters in public healthcare and among individuals involved in health policy-making across European countries. Such knowledge may be of vital importance to be able to fully grasp and handle risks associated with use of CAM on an individual and structural level, both within national public healthcare systems and cross-border.

The research questions are:

1. How is “risk” understood by individuals involved in health policy-making and clinical encounters across the two included studies?
2. How might these risk understandings influence different stakeholders’ decision-making, risk communication, and health policy-making with regard to CAM use within and across European countries?

## Methods

### Material

This empirical analysis was based on two already introduced qualitative studies with materials that include different stakeholders’ perspectives on risk understandings and patient safety [14, 15, 28–34]. The studies are inter-related because they had open-ended, qualitative designs and data turned out to include information on different stakeholders’ understandings and handling of risks associated with the use of CAM. In the EU-study CAMbrella, documents and personal communications dealing with legislation and regulation of CAM were collected from the European Union (EU) and from 39 European countries [30–32]. The general legal and regulatory status of CAM on the first and second national legal level was reviewed, including supervision and reimbursement. Data on the regulation of 12 CAM treatment modalities and CAM providers/professions in each of the 39 countries were also collected. The material is extensive and consists of a great number of legal documents and statements from official web-sites and government documents dealing with regulation. Personal communications consist of answers to questionnaires and interviews, in addition to notes and minutes from telephone conversations, emails, personal meetings and conference discussions. Persons included in personal communications were representatives for Ministries of health, law and education, governmental representatives, CAM providers, members of CAM associations, and researchers in the 39 included countries (for further information, see 30–34). In the study on understandings of benefits and risks associated with the use of CAM, in-depth interviews with 31 Norwegian and Danish patients who were CAM users and diagnosed with cancer (19 participants) or multiple sclerosis (12 participants) and 12 of their doctors (5 physicians, 4 oncologists, 3 neurologists) were conducted [14, 15, 28, 29]. The main themes in the CAM user interviews were personal history, being a patient within the public health-care system, experiences from doctor–CAM user communication, and reasons for the choice and use of CAM including understandings of risk and risk assessment. In the interviews with doctors, the main issues concerned experiences with patients’ CAM use, risk understandings associated with conventional medicine and CAM, and doctor-CAM user communication. The interviews lasted from 45 to 150 min, and were conducted by the first author and an experienced research assistant [14, 15, 28, 29].

### Methods

In-depth individual interviews and document analysis were selected as research methods in the two included studies. These qualitative methods were chosen because we explored under-researched and experience-based issues of which there were little previous knowledge [35]. It has been strongly argued that CAM research demands methodological approaches that incorporate subjectivity, everyday life, environment, and the complexity of modern health-care systems. The validity of qualitative methodology has thus been identified as fundamental to understanding and describing the philosophical basis, key treatment components, and contextual frameworks of CAM use and CAM modalities [24–26], here including risk understandings and health policy-making.

We determined qualitative content analysis to be a suitable analytical approach, defined as a systematic classification process and identifying different themes or patterns, and used to interpret meaning from the content of text data [36]. Furthermore, we compared and integrated the original findings from the two different, yet inter-related studies. Through the construction of a case history, the findings were further described and analyzed. Thus, the technique had an interpretive, rather than aggregating intent, in contrast to meta-analysis of quantitative studies which is aggregative and reduces data to a single unit [37, 38]. Because the authors themselves have conducted the included studies, and also have been working together in a multi-disciplinary research group focusing on patient safety, we claim that the interpretations in the comparison of study results acknowledge the intent and philosophical basis for each of the original studies [38]. In a final analytical step, the authors developed a table of questions to ask in settings possibly involving differing risk understandings that may influence patient safety nationally and cross-border. We have chosen to present the main themes and patterns revealed across the two studies as the constructed case *Sara* instead of presenting an analysis based on quotations from interviews and personal communications. This approach was chosen because a case can visualize the important contextual and processual aspects of the empirical material with respect to our research questions [39, 40]. We thus demonstrate the importance of acknowledging stakeholders’ differing risk understandings in regulation/health policy-making and clinical settings involving use of, and/or discussion about the use of CAM, within a real-life context. Constructed cases are often used as exploratory, descriptive and explanatory research and to generate theory and initiate change in practice and education [38, 39]. The case *Sara* is based on the experiences and reflections of a real cancer patient participating in one of our studies among CAM users and their medical doctors, and combined with important empirical patterns revealed in multiple individual interviews with different stakeholders in the CAMbrella study [14, 15, 28–34]. Some quotations from the in-depth interview with this patient are included in the case presentation, however with several changes made to include important information from the CAMbrella study. In this constructed case, we have also added use of acupuncture in Norway and abroad to the treatments *Sara* actually used, because the regulation of acupuncture in Europe is a striking example of disharmonized regulation in European health care [30, 32, 34].

### Ethics, consents and permissions

The interview study was approved by the Regional Committee for Medical and Health Research Ethics North (P REK NORD 28/2005), and the Norwegian Centre for Research Data (project number 13409). In the CAMbrella project, interviewees were asked to confirm the author’s comprehension of their answers, and if cited, to confirm the use of their answers with reference to their names, position and/or rank [30].

## Results

When comparing the two studies, it was not possible to identify a “CAM treatment safety system” based on clinical and regulation standards, professional training, definitions and terminology. In a secondary analysis of the CAMbrella material, we found that different government and public sector employees within and between countries answered the same questions very differently. We found the same patterns in the answers from providers either representing CAM associations or themselves as CAM health professionals or providers. We found that, even within the same ministry, government department, institution or hospital, descriptions and specifications related to providers, treatment standards and regulations were interpreted differently among the involved persons**.** In legislation and legal preparatory work it is thus difficult to settle if, and eventually how, individual incoherent risk understandings have been taken into consideration. Arguments for and against CAM regulation, in a risk and patient safety perspective, are very difficult to structure and compare. In a secondary analysis of the interviews with CAM users and medical doctors, the same lack of common risk understandings and regulatory systems was identified. The CAM users found CAM to be “green”, “natural” and “safe”, while their medical doctors found no evidence to trust CAM treatments. Neither the CAM users nor the medical doctors had been previously asked about their risk understandings, and the rather fundamental gaps between risk understandings related to CAM use in these two groups negatively influenced risk communication. Different stakeholders’ unclear and differing risk understandings across the two studies seemed to be influenced by a variety of factors involving education, individual experience from professional and personal settings, cultural beliefs, social practices, attitudes, and values. In the following constructed patient/CAM user case, we further demonstrate these results on different aspects of gaps in risk understandings and their possible consequences as revealed across the two materials. The results are summarized after the case presentation, as a basis for an in-depth discussion of their possible implications.

### “Then I went to a hospital abroad”: Sara’s cancer trajectory in a European patient safety perspective

Sara is a nurse in her 50’s, with 30 years of experience from working with cancer patients in Norwegian hospitals. Soon after being diagnosed with breast cancer stage II in 2010, she decided to combine conventional treatment offered in the Norwegian public health-care system with CAM treatments found outside this system. Her decision was based on a personal knowledge basis consisting of both experiences such as the observation of dramatic, fatal cancer courses in her family and in hospital settings, and scientific knowledge about adverse effects from conventional cancer therapies. Furthermore, Sara was familiar with and believed in CAM therapies after having taken an education in reflexology herself. She experienced that her focus on bodily experiences and personal efforts, which she found to be crucial for a successful treatment of her cancer, was not accepted within the Norwegian public health-care system.

Sara received surgery and suffered through a difficult first period of chemotherapy that caused severe adverse effects. After that, she perceived continued conventional cancer treatment as a considerable risk to her health and quality of life at that point of her cancer trajectory. She collected a lot of information about treatment options, and then decided to use different alternative therapies provided by CAM practitioners. Sara explained:

*I had faith that there are many roads to Rome… I thought that CAM is natural and would not harm me… Most people having had similar surgery experience severe adverse effects. I didn’t want to poison the body with chemotherapy and radiation. I had good reason (based on knowledge of conventional and alternative treatment options, authors’ comment) to believe that I could reach a better goal without continuing the conventional treatment at the moment.*

After a period of self–care, Sara decided to visit a hospital in another European country. In this hospital, cancer patients learned to manage their cancer from a therapeutic perspective that included both body and mind. The hospital had Western medically educated doctors and was part of the public health-care system in the country where it was located. All treatments, such as acupuncture and herbal medicine, were regulated within the public health-care system. Despite this, Sara experienced that the Norwegian hospital doctors were fundamentally skeptical to the medical skills of their foreign colleagues, and the possible risks associated with the treatments Sara wanted to use. Several of these treatments were defined as CAM according to Norwegian legislation. Among these treatments were herbal medicine and a certain acupuncture treatment that Sara thought would strengthen her immune system and thus possibly enable her to receive chemotherapy in the Norwegian hospital later on. According to Sara, the Norwegian doctors were unwilling to discuss her treatment decisions. They were fundamentally skeptical because they found only limited scientific evidence for positive outcomes or an acceptable level of risk associated with the treatments Sara wanted to use. Based on information from the foreign hospital and the Internet, and discussions with friends, Sara found that the CAM treatments were safe and would have no negative influence on the conventional cancer treatment she planned for in Norway when she returned home. Sara eventually went to the hospital abroad, and was very satisfied with the treatments, the treatment philosophy and the health-care professionals she met:

*The treatments were very holistically oriented, everything from diet and nutrition to focus on patients’ mental health status, stressful lives and so forth… The doctors were all so nice and friendly and remembered all the patients’ names…The same doctor visited me every day, and I was told that I could ask to see her anytime. When I told her about the skeptical Norwegian doctors, she said, very friendly, that she thought their attitude would change over time towards a more holistic medicine, based on natural products and focus on mind, body and self-care.*

According to *Sara*, she was not offered a check-up at the Norwegian hospital after she returned home and she has not received any conventional cancer treatment since then. *Sara* still feels abandoned by the Norwegian hospitals’ oncology department, but has established a very well-functioning relationship with a general practitioner in her village and relates to his medical advice.

### Summary of results

By combining and comparing individual and structural perspectives on risk, CAM use and regulation, we have revealed that there are underexplored gaps in risk understandings among individuals involved in European CAM regulation and legislation, and between CAM users and their medical doctors. The constructed case *Sara* demonstrates a lack of harmonized risk understandings between health policy makers and health professionals in different countries, and between both these groups of stakeholders and *Sara* as a patient and CAM user. Furthermore, the case demonstrates a lack of information and communication on risk and patient safety associated with CAM use and cross-border health care. Consequently, patients/CAM users, public health-care providers, CAM providers, health authorities and researchers may have insufficient information to understand and assess risk in settings involving CAM. On an individual level, this is a potential problem between the public health-care system and the patient/CAM user, e.g., if health professionals refuse to discuss CAM use with a cancer patient or a patient/CAM user who do not dare to raise the issue. Also on a structural level, this case demonstrates possible gaps in risk understandings that might negatively influence patient safety and the risk scenario for *Sara*. The CAM treatments she chose to use were subject to rather different public regulation in the two countries involved, and the claim, reimbursement and supervision systems between the two countries differed fundamentally. In some countries, CAM modalities and providers are regulated with educational requirements, treatment standards and ethics, within the same system as conventional public health-care services. Interviewees from these countries underlined that regulation is important to be able to perform supervision of CAM providers, secure patient’s claim rights, and control safety aspects like treatment quality assessment and safety information to patients like *Sara*.

## Discussion

According to the EU Directive 2011/24/EU on the application of patients’ rights in cross-border health care [41], patients like *Sara* have the right to be informed. To make it possible to provide and receive correct information about different treatment options across national borders, there has to be a common understanding of facts, regulation and terminology on one hand, and treatment and provider requirements on the other. Consequently, we could ask; who “owns” the correct descriptions of facts when there are few common understandings of safety and risks issues among different stakeholders such as *Sara*, the Norwegian hospital doctors and the doctors working in the foreign hospital?

### Regulation and educational background for CAM providers in cross-border health care

**Fig. 1:**
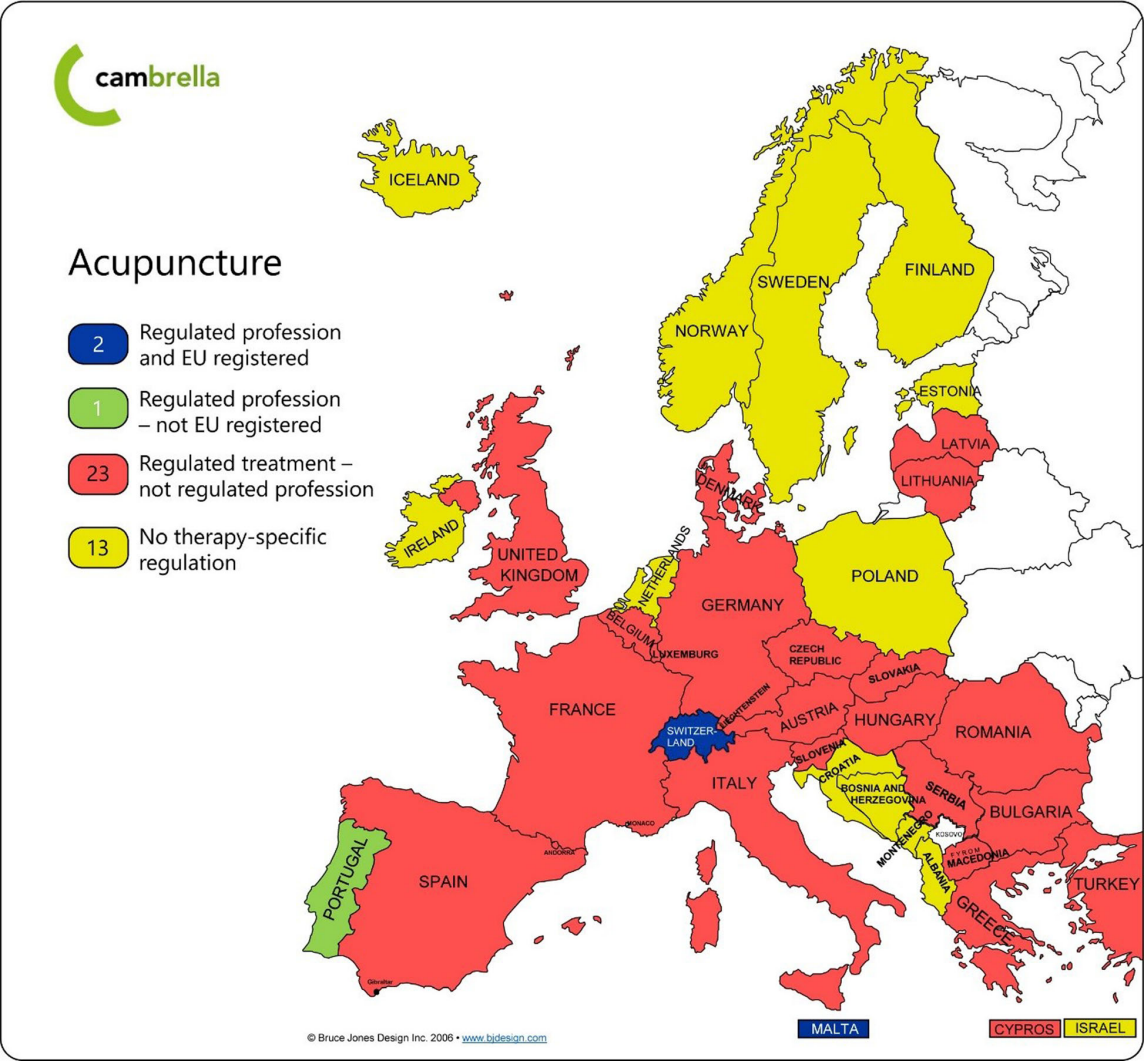
“Regulation of Acupuncture in Europe” depicted in Fig. 1 has been developed by the second author Wiesener, S and this updated version has not been published before. The map is developed on the basis of an uncolored clip-art map of the European countries, retrieved from: “Bruce Jones design Inc. 2006 - http://cam-regulation.org/en/acupuncture-map”. The clip-art map was purchased first time for use by NAFKAM in the CAMbrella project FP7-HEALTH-2009, GA No.241951. NAFKAM allows the authors to develop maps using this purchased clip-art

In a first setting, *Sara* may receive acupuncture in the Norwegian hospital, provided by an authorized, “regulated” medical doctor or nurse. In another setting, *Sara* chooses to use an acupuncturist outside the regulated Norwegian public health-care system. The acupuncturist will then have an unregulated, and often unknown, training and capability of handling possible risks associated with the treatment. An important question is whether *Sara* is less at risk when receiving acupuncture treatment at the hospital by a “regulated” medical doctor or nurse than she is when receiving acupuncture by the “unregulated” acupuncturist? In Norway, this acupuncturist is probably a member of an established acupuncture association. These associations have claims to their members about medical education, acupuncture training and ethics. Concerns have been raised about the quality of acupuncture training among Norwegian nurses and medical doctors who provide acupuncture within the public health-care system [42]. However, if *Sara* receives acupuncture within the public health-care system the Norwegian authorities can support her with a legal basis for compensation of possible health damages, reimbursement of costs and supervision of the involved health professionals. If *Sara* chooses the “unregulated” acupuncturist, she does not have these rights. In a third setting, *Sara* decides to receive acupuncture in another European country than Norway. Which country she chooses to go to, may strongly influence her rights as a patient. Acupuncture is regulated in 26 out of 39 European countries [30, 32], as shown at the updated CAM regulation website from National Research Center in Complementary and Alternative Medicine (NAFKAM) in Fig. 1 based on data from 2017 [32].

When checking the details in each country, we find that none have similar legislation. Furthermore, we find that in three countries, “acupuncturist” is a regulated health profession, while in 23 countries “acupuncturist” is not a regulated profession, only the acupuncture treatment itself is regulated. The last 13 countries have no specific acupuncture treatment regulation, but may be covered by the general health regulation. In some of the European countries, only doctors may provide acupuncture treatment. The educational level to be approved to provide acupuncture differs from courses offered by an acupuncture association (e.g., Cyprus) to 3–5 years of university training (e.g., Hungary) [30, 32]. We know that CAM users favor CAM providers that are governmentally authorized health professionals [43]. However, *Sara* will probably not know the qualification requirements for a medical doctor to get approval to practice acupuncture, his/her actual training in acupuncture, the reimbursement rights in the actual country or how to make claims if the acupuncture treatment causes harm. If a patient like *Sara* crosses borders between countries to receive a treatment, both language, culture and other contextual factors may make important information on patient safety difficult to access and understand. Patients who choose CAM treatments are thus often not able to be “informed patients” in cross-border health care as described in the EU Directive 2011/24/EU [41]. If *Sara* receives acupuncture treatment in Hungary, the provider will probably be a medical doctor with an additional acupuncture degree from a university. If she chooses to receive acupuncture in Ireland, where CAM is unregulated, the provider may be either a non-professional or a health-care professional. As clearly demonstrated by these examples, the situation is complex in several important aspects, which may all represent a possible threat to patient safety for CAM users in national and cross-border health-care settings.

### Communication about risk and possible implications for patient safety

Some patients, e.g., *Sara*, decide to delay or decline offers of conventional treatment, and use CAM instead. In concordance with *Sara*’s case, studies have revealed that such treatment decisions often represent a huge challenge in risk communication between doctors and cancer patients [14, 15, 28, 29, 44]. Their understandings of risks and benefits associated with CAM and conventional cancer care are often incompatible in situations where cancer patients choose to delay or decline conventional medicine*.* These patients challenge the rationality of medical advice and the authority of oncology experts, and may be subject to risks associated to several aspects of CAM use [13–16, 28, 29]. In worst cases, this may have fatal consequences. Studies of clinical outcomes of decliners of conventional cancer care have revealed that failure to comply with the public health-care system and the recommended conventional care led to increased risk of cancer progression and/or death [45, 46]. If health professionals like those at the Norwegian hospital refuse to discuss CAM use and cross-border health care with their patients, this may negatively influence the patients’/CAM users’ trust in public health care, and may function as a push-factor for the patient/CAM user to delay or decline conventional treatment. Although *Sara* wanted to use alternative treatments and postpone chemotherapy and radiation, she really wanted to uphold her relationship with the public health-care system. This is the situation with most CAM users [14, 19–21]. It signals that if the public health care wants to be trusted and actually act person-centred [47], it is of crucial importance to focus on well-functioning communication and increased knowledge about CAM users’ perspectives on benefits and risks associated with the use of CAM and conventional treatment [13, 14, 20–24, 48, 49].

### The importance of acknowledging differing risk understandings

As noticed in the introduction, the ontological status of risk is heavily debated within the social sciences [1, 2, 22, 23, 27]. Approaching risk from various methodological perspectives is important also in medical discourses as there may exist a “…significant problem with using epidemiological risk assessment, [which] is that risk is reduced to a statistical measure that does not take into account the attitudes or risk-taking behaviors of human beings” ([22], p. 66). Based on the comparison of the studies under investigation in this article, we argue that this argument raised with regard to studies of risk in conventional medicine and public health care may be *even more relevant* for measurement and communication of risks associated with the use of CAM. This does not mean that CAM treatments necessarily are associated with higher levels of risk than conventional treatments. Our argument is that the use of CAM, in a risk perspective, is linked to *uncertainty* caused by factors like gaps in risk understandings among different stakeholders, disharmonized regulation across countries, lack of scientific evidence and insufficient communication. Thus, risk definitions related to the use of CAM should include elements of uncertainty and subjectivity, as argued in the introduction [1, 2, 22, 23, 27]. This study and our previous studies have brought to attention a number of questions and challenges regarding risk, CAM use and regulation. We thus call for a comprehensive and extended understanding of risk factors associated with the use and regulation of CAM. To develop more comprehensive risk understandings across stakeholders, national borders and treatment contexts, we have transformed the findings revealed in this study into questions to ask in various practical settings.

### Introducing questions to ask for policymakers, in clinical settings and in risk research that incorporate patients’ use of CAM

To transform the findings of this study into practical settings and contribute to more comprehensive risk understandings in regulation, clinical practice and research involving European patients’ use of CAM, we hereby introduce four sets of relevant questions to ask in different settings involving various stakeholders (Table 1). These sets of questions do of course not cover all situations, but may function as a starting point that can be further developed to fit specific settings involving risk understandings and risk definitions.

**Table 1:** Key questions to ask for policymakers, in clinical settings and in risk research that incorporate patients’ use of CAM

Settings	Questions
Regulation - the policy-makers perspective	• Which decision factors (like research, efficacy and patient safety elements) is the national regulation of CAM based on? • How should scientific evidence on direct and indirect risk factors influence regulation of the specific CAM modality? • Which provider training and treatment requirements will strengthen the safety for CAM users/patients? • How may a planned regulation of CAM providers and treatments increase patient safety? • How can more harmonized and integrated regulation of conventional medicine and CAM result in increased patient safety? • How should we emphasize safety aspects of European regulation on CAM when deciding new CAM regulation on a national level? • How will the considered regulation of CAM influence public supervision possibilities and patients’ claim and reimbursement rights?
Clinical settings– provider perspective	• How does this CAM user understand risks associated with conventional treatment? Why? • How does this CAM user understand risks associated with CAM/specific CAM treatments? Why? • How do I myself as a health-care provider understand risk associated with CAM and conventional treatment? • Do my risk understandings differ from those of the patient in front of me? • How can I best communicate important risk information to this patient?
Clinical settings– patient perspective	• How do I as a patient perceive my personal risk associated with the use of the CAM I want to use? • How do I as a patient perceive my personal risk associated with the use of the recommended conventional treatment? • How can I best communicate my interest in CAM to medical doctors and nurses in public health care? • What does this doctor know about CAM? • What training does this CAM provider have? • What does this CAM provider know about medicine/conventional treatment? • Where can I find trustworthy information about CAM treatments and CAM providers? • Is the CAM treatment I want to use associated with any possible risks? • Is the conventional treatment I use associated with any possible risks? • Are there any known risks associated with the combination of the conventional and the CAM treatment I want to use? • How can I deal with possible risks?
Risk research	• Does the risk study we are planning include an understanding of risk as an objective or a subjective phenomenon? • Have we actually defined the concept of risk in our study? • Would another risk understanding/definition influence the study in terms of research questions, methodology, and interpretation of results?

## Conclusion: we must confront the question “What is risk?”

Many citizens in European countries combine conventional treatment with CAM, and it is thus of crucial importance to acknowledge the possible existence and implications of differing risk understandings across stakeholders and countries. By combining and comparing individual and structural perspectives on risk, CAM use and regulation, this study has demonstrated that different stakeholders may understand and handle risk very differently. We argue that this situation may have a negative and so far underestimated influence on patient safety for European CAM users. Any attempt to regulate, communicate about or study risk in CAM should thus confront the question “*What is risk?*”. The suggested key questions in Table 1 may contribute to operationalize this fundamental question in different settings. In health care policy-making, answers to this question may strongly influence public health care regulation. In clinical practice, such answers may represent new knowledge that may contribute to better communication and improved patient safety for CAM users. In research contexts, answers to this question may have implications with respect to research questions, methodology, what aspects of risk that are revealed, and the interpretation of study results.

## Abbreviations

CAM: Complementary and alternative medicine
EU: European Union
NAFKAM: The national research center in complementary and alternative medicine
